# Effect of Probiotics on the Performance and Intestinal Health of Broiler Chickens Infected with *Eimeria tenella*

**DOI:** 10.3390/vaccines10010097

**Published:** 2022-01-10

**Authors:** Muhammad Mohsin, Ziping Zhang, Guangwen Yin

**Affiliations:** 1 College of Life Science, Fujian Agriculture and Forestry University, Fuzhou 350002, China; onlymohsindvm@gmail.com; 2 College of Animal Sciences (College of Bee Science), Fujian Agriculture and Forestry University, Fuzhou 350002, China; 3 Key Laboratory of Marine Biotechnology of Fujian Province, Institute of Oceanology, Fujian Agriculture and Forestry University, Fuzhou 350002, China; 4 Department of Parasitology, University of Agriculture, Faisalabad 38000, Pakistan

**Keywords:** *Eimeria tenella*, probiotics, immunity, gene expression, poultry

## Abstract

Coccidiosis is an important parasitic disease of poultry with great economic importance. Due to drug resistance issues, the study was conducted to investigate how probiotics (*Lactobacillus plantarum or L. plantarum*) affected oocysts per gram of feces (OPG), fecal scores, feed conversion ratio (FCR), immunomodulatory effect in terms of the cell-mediated and humoral immune response. Serum chemistry (ALT, AST, LDH, and creatinine) was measured in different treated chicken groups. mRNA expression levels of antioxidant enzymes (SOD 1 and CAT), peptide transporter 1 (PepT 1), and tight junction proteins (ZO and CLDN 1) were also examined in chicken groups infected with *Eimeria tenella (E. tenella).* Chickens supplemented with *L. plantarum* 1 × 10^8^ CFU (colony-forming unit) showed an improved cell-mediated and humoral immune response, compared with the control group (*p* < 0.05). Probiotics also enhanced the performance of antioxidant enzymes, PepT 1, and tight junction proteins, and improved serum chemistry (AST, ALT, and LDH), compared with control-infected, non-medicated chickens. However, no significant difference (*p* > 0.05) was observed in CLDN 1 expression level and creatinine in all treated chicken groups. These findings demonstrated that probiotics supplementation in the feed can protect the birds against *E. tenella* infection.

## 1. Introduction

Intestinal diseases of parasitic origin constitute a major problem for domestic poultry and animals worldwide [1,2,3,4]. The phylum Apicomplexa contains numerous well-known obligatory and intracellular parasites of humans and livestock. In these phyla, the genus *Eimeria* is well known and is the cause of coccidiosis in birds and mammals [5]. Avian coccidiosis is an enteric disease of protozoal origin that causes malabsorption, bloody diarrhea, poor feed conversion ratio, retarded growth, and increased mortality [6,7]. It also enhances the susceptibility of birds/animals to various other enteric diseases, including necrotic enteritis. According to an estimate, annual losses to the poultry industry (USD 3 billion) are a well-established fact [8]. These losses are associated with high morbidity, medicinal expenditure, and birds mortality [9,10]. The chickens are affected by seven species of *Eimeria* parasite; however, *E. tenella* is considered the most pathogenic parasite among them. It is associated with the destruction of the cecal epithelium and is also responsible for severe disruption of intestinal homeostasis [3,11].

Live vaccines and various anticoccidial drugs are commonly used to control coccidiosis [2,10]. However, numerous issues are associated with anticoccidial drugs, among which are drug resistance and drug residues in food products in different world regions [2,12]. Due to these problems, alternative strategies for controlling coccidiosis are needed for this age [13]. Probiotics are considered safe and appealing alternatives in controlling enteric pathogens, including *Eimeria* parasites [14,15]. They can also compensate for the various issues related to the use of anticoccidial vaccines and drugs [3,6].

Probiotics are non-pathogenic microbes that can maintain normal intestinal microbiota and positive activity against intestinal diseases [16]. Probiotics supplementation in the poultry feed enhances the activity of beneficial microbes in the gastrointestinal tract (GIT) [14], increases the performance of digestive enzymes, stimulates the humoral and cell-mediated immunity [4,14], and also neutralizes various enterotoxins [17,18]. Probiotics positively affect the blood parameters, carcass traits, intestinal microflora, growth, and immune functions of the birds [19]. Probiotics can act as anticoccidial agents, with immunomodulators such as improved Toll-like receptor expression [20], cytokine stimulation [21], antibody development [15,22], antioxidant effect [23,24], lower lesion score [4], decreased oocysts shedding [3,25], less fecal score [14], as well as neutralizing various enterotoxins. Based on these results, probiotics have been considered as potential anticoccidial agents; however, there is limited confirmation [26].

The immune system against avian coccidiosis could be categorized as innate and adaptive [27]. The innate immune response (first line of protection) provides the recognition of pathogen-associated molecular patterns (PAMPs) retained by pattern recognition receptors (PRRs), such as Toll-like receptors (TLRs) [14,28]. These receptors (ligand) contribute to the activation and proliferation of cytokines and immune cells. Macrophages, epithelial cells, dendritic cells, natural killer cells, and heterophils are the main cells that contribute against avian coccidiosis in the innate immune response. However, the adaptive immune response is specific to the antigen. It stimulates the two main kinds of lymphocytes—B cells (immunoglobulins production) and T cells (T-cell receptors), against pathogens, including *Eimeria* parasites [29]. The objective of the manuscript is to provide a comprehensive explanation of the use of probiotics in chicken farms to combat coccidiosis in the feed and their effects on mRNA gene expression as growth promotor, immunomodulatory, and serum chemistry of chickens.

## 2. Materials and Methods

### 2.1. Ethics Statement

All experiments using chickens were carried out in compliance with the recent Chinese Ethical legislation, and special attention was given to the welfare of experimental chickens. The animal protocol was accepted by the Animal Care Committee of Fujian Agriculture and Forestry University (FAFU2021-3-12).

### 2.2. Parasite Propagation

**Figure 1:**
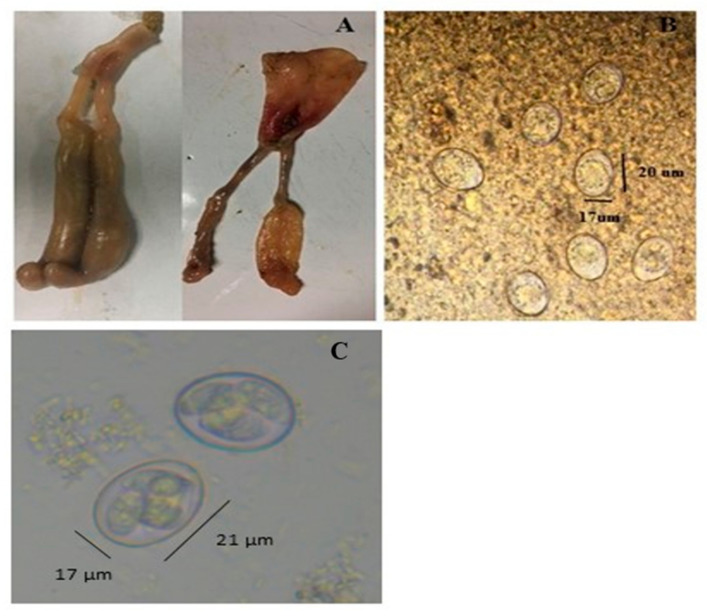
Collection and propagation of different *E. tenella* life stages from the infected caecum of the chicken: (**A**) *Eimeria tenella*-infected caecum of 14-day old chicken; (**B**) under the microscope; unsporulated oocysts (from fecal sample of the infected chicken) are denoted by arrows with a width of 17 μm and a length of 20 μm; (**C**) sporulated oocysts (converted from unsporulated oocysts to sporulated oocysts) observed under the microscope, denoted by arrows with 17 μm width and 21 μm.

*E. tenella* (Beijing Strain) was provided by Professor Xun Suo in China Agricultural University, then maintained and propagated in our laboratory with the following steps: First, 200 one-day-old chickens were grown in different cages; feed and water were provided ad libitum in hygienic cage systems. On day 14, chickens were infected with *E. tenella* (3 × 10^4^ sporulated oocysts/bird) through the oral route. The chicken infected with *E. tenella* typically showed the cecal lesion (Figure 1A). Unsporulated oocysts were collected (5–9 days of post infection) from infected bird droppings (Figure 1B) and converted to sporulated form (Figure 1C), following Yin et al. [30]. The steps for the collection of un-sporulated and sporulated oocysts were as follows: In detail, on the 5th day of infection, the dropping pan was emptied, and the chickens’ droppings were collected during days 6 to 9 of infection. Infected bird droppings were poured into the clean water and mixed well after filtering the mixture (droppings and water) by using a sieve. The filtrate was centrifuged at 3500 r/min for 5 min, and the supernatant was discarded. Saturated sodium chloride solution (NaCl) was poured into the precipitate and mixed thoroughly and again centrifuged at 3500 r/min for 5 min, after which the supernatant was collected. Then, 3–4 volume of ultrapure water was added to the collected liquid and centrifuged at 3500 r/min for 15 min, and the precipitate was collected. Next, 8–12 times the volume of 2.5% potassium dichromate solution (K_2_Cr_2_O_7_) was added to the precipitate, stirred evenly, and placed in a shaker at 28 °C and 120 rpm speed for 2 days (for unsporulated to sporulated oocysts stage of the parasite). After two days, the sporulation status of coccidial oocysts was observed, the number of oocysts was counted under a microscope using a McMaster chamber, and afterward, they were stored in a refrigerator at 4 °C for later use.

### 2.3. Experimentally Treated Chicken Groups

Chickens were raised in different cages. On day 14 of the trial, chickens (*n* = 120) were placed into 5 different groups (24 chickens per group) and each group was divided into three cages per treatment (8 chickens per cage). Feed and water were provided ad libitum. During the first week of age, the temperature was maintained at 30–32 °C; however, it was reduced on weekly basis by 2–3 °C and maintained at 23–25 °C through the end of the experiment (42 days). Group PROB6 was infected and supplemented with *L. plantarum* 1 × 10^6^ CFU in the feed; group PROB7 was infected and supplemented with *L. plantarum* 1 × 10^7^ CFU in the feed; group PROB8 was infected and supplemented with *L. plantarum* 1 × 10^8^ CFU in the feed; group NC (infected but no additive control) was the infected, non-medicated group; group Cont (naïve control) was taken as the non-infected and non-medicated control group. Groups excluding group Cont were infected with *E. tenella* (3 × 10^4^ sporulated oocysts) through the oral route (day 14 of trial). Here, groups NC and Cont were kept as control groups.

### 2.4. Oocyst per Gram (OPG) and Fecal Score Assessment

The oocyst per gram was assessed on 8, 10, and 13 days post infection of chickens (d.p.i.). Two grams (g) of feces was collected and crushed from each replicate. The crushed fecal sample was placed in a glass beaker (250 mL), and 58–60 milliliter (mL) of saturated sodium chloride (NaCl) solution was added and thoroughly mixed. The prepared mixture was left for 5–10 min before the count, allowing the oocysts to float to the surface. Finally, samples from each replication were collected and checked under the microscope using the McMaster chamber [31]. After 5, 8, and 13 days of *E. tenella* infection, the anticoccidial potential of all groups was evaluated using the fecal score (from 0–4), following Youn et al. [32].

### 2.5. Feed Conversion Ratio

The following formula was used to evaluate the feed conversion ratio (FCR) for all experimental groups during 42 days of the research:

Feed conversation ratio (FCR) = Mean feed consumption/Mean weight

### 2.6. Tissue and Serum Collection

On day 8 of post infection, six average birds/group were slaughtered. The cecal samples were collected for mRNA gene expression and kept at −80 °C until further use. On days 7 and 14 after the injection of sheep red blood cells (SRBCs; non-pathogenic T-dependent antigens), blood samples were collected from chicken wing vein for serum extraction, following Adamu et al. [29]. Microplate hemagglutinin assay was used to quantify the anti-SRBC antibody titers. Serum samples were used for the total Ig and IgG against SRBCs.

### 2.7. Immune Response

#### 2.7.1. Cell-mediated Response to Dinitrochlorobenzene (DNCB)

DNCB test was used to evaluate the delayed-type hypersensitivity reaction, following Blumink et al. [33]. Briefly, the first dose of 0.1 mL (2% DNCB in acetone) was administered on the 4 cm^2^ area on the skin of the five chickens/group (day 14), followed by the second dose on day 21 of the experiment. Skin thickness was measured using a vernier caliper before and after 24 h of DNCB administration.

#### 2.7.2. Humoral Immune Response to Sheep Red Blood Cells (SRBCs)

Humoral immune responses were analyzed in total immunoglobulin (Ig) and IgG against SRBCs (non-pathogenic T-dependent antigens). Microplate hemagglutinin assay was used to quantify the anti-SRBC antibody titers by following the method in [25]. Briefly, 1 mL SRBCs suspension was injected through the intramuscular route on day 14 (parasite infection day). Serum was collected on days 7 and 14 after SRBC injection. Serum samples were analyzed for total Ig and IgG anti-SRBCs antibodies, and results were shown as geomean titers (GMT).

### 2.8. Serum Chemistry

Serum chemistry included aspartate aminotransferase (AST) and alanine transferase (ALT). Creatinine and lactate dehydrogenase (LDH) values were measured following the diagnostic kit instructions (Bioengineering Institute, Nanjing, China).

### 2.9. RNA Extraction, cDNA Preparation, and Determination of mRNA Gene Expression

The total RNA was extracted from the infected caecum using the kit method (Omega Bio-tek, Guangzhou, China). RNA pellets were dissolved in RNase-free water (adequate RNA concentration), and the RNA concentration was measured spectrophotometrically (absorbance at 260/280 nm) and kept at −80 °C until further use. Conversion of mRNA to cDNA was completed following the kit instruction (Yeasen Biotech, Shanghai, China). The mRNA expression levels of antioxidant (SOD, CAT), PepT 1, and tight junction protein (CLDN 1, ZO-1) were measured by qPCR. The primers used in the research are shown in Table 1. SYBR Green Supermix (Bio-Rad, Nanjing, China) was used for qPCR on a Roche Light Cycler 480 Real-Time System. The following were the qPCR conditions: after initial denaturing for 30 s at 95 °C, 40 cycles of 10 s at 95 °C, and 20 s at 60 °C were programmed.

### 2.10. Statistical Analysis

Analysis of variance was used to examine data obtained from various parameters. Mean values were compared by Tukey’s test using Statistix software (San Jose, CA, USA). Statistical differences among group means were considered significant at *p* < 0.05.

## 3. Results

### 3.1. Fecal Score and OPG Assessment

**Figure 2:**
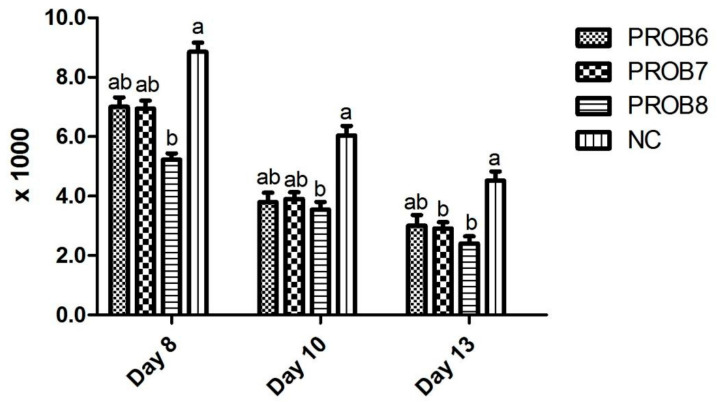
Oocysts per gram (OPG) of feces with experimentally induced coccidiosis in various treatment groups at day 8, 10, and 13 post infection. Means with different superscripts (a, b) are significantly different (*p* < 0.05). X-axis represents the numbers of different groups, and y-axis shows the numbers of oocyst in the chicken dropping. PROB6 = *L. plantarum* (1 × 10^6^ CFU) supplemented group in feed; PROB7 = *L. plantarum* (1 × 10^7^ CFU) supplemented group in feed; PROB8 = *L. plantarum* (1 × 10^8^ CFU) supplemented group in feed; NC = infected, non-medicated treatment group; Cont = non-infected, non-medicated group.

Fecal score and OPG values in chicken groups supplemented with probiotics are shown in Table 2 and Figure 2. A non-significant difference was observed (fecal scores) in *L. plantarum* supplemented groups (A and B) in feed. However, a significant difference (*p* < 0.05) was observed in probiotic-supplemented groups (1 × 10^8^ CFU) on 5, 6, and 8 days post infection, compared with the infected, non-medicated control group. OPG was assessed in *L. plantarum* supplemented groups on 8, 10, and 13 days post infection (Figure 2). Fewer OPG was observed in the probiotic-supplemented group (1 × 10^8^ CFU) in feed than in infected, non-medicated control groups, as shown in Figure 2.

### 3.2. Feed Conversion Ratio

**Figure 3:**
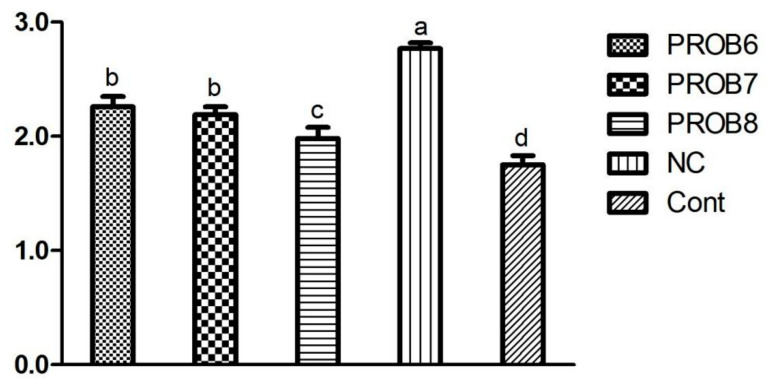
Feed conversion ratio (FCR) in chickens with experimentally induced coccidiosis in different treated groups. Means with different superscripts (a, b, c, d) are significantly different (*p* < 0.05). PROB6 = *L. plantarum* (1 × 10^6^ CFU) supplemented group in feed; PROB7 = *L. plantarum* (1 × 10^7^ CFU) supplemented group in feed; PROB8 = *L. plantarum* (1 × 10^8^ CFU) supplemented group in feed; NC = infected, non-medicated treatment group; Cont = non-infected, non-medicated treated group.

*L. plantarum* supplemented groups in feed showed a better FCR than the *E. tenella* infected control group, as shown in Figure 3.

### 3.3. Cell-Mediated Immune Response

**Figure 4:**
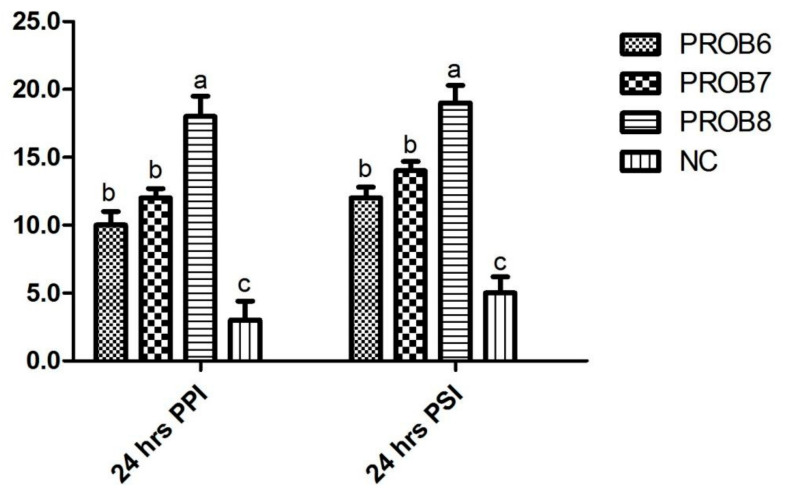
Cell-mediated response to DNCB in different probiotic-supplemented and control groups. Means with different superscripts (a, b, c) are significantly different (*p* < 0.05). PROB6 = *L. plantarum* (1 × 10^6^ CFU) supplemented group in feed; PROB7 = *L. plantarum* (1 × 10^7^ CFU) supplemented group in feed; PROB8 = *L. plantarum* (1 × 10^8^ CFU) supplemented group in feed; NC = infected, non-medicated control group.

Improved cell-mediated immune response to DNCB was observed in all probiotic-supplemented chicken groups (*p* ˂ 0.05), compared with that of the infected, non-medicated control group. Among probiotic-supplemented groups, the maximum cell-mediated response was noted in chickens supplemented with 1 × 10^8^ CFU, followed in decreasing order by the groups supplemented with 1 × 10^7^ CFU and 1 × 10^6^ CFU (Figure 4).

### 3.4. Humoral Immunity

**Figure 5:**
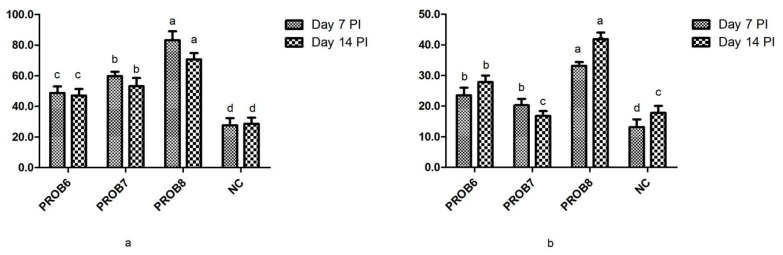
Humoral response to SRBCs in different probiotic-supplemented and control group: (**a**) total immunoglobulin (Ig) at 7 and 14 days post infection (PI); (**b**) IgG assessment at 7 and 14 days post infection (PI). Means with different superscripts (a, b, c, d) are significantly different (*p* < 0.05). PROB6 = *L. plantarum* (1 × 10^6^ CFU) supplemented group in feed; PROB7 = *L. plantarum* (1 × 10^7^ CFU) supplemented group in feed; PROB8 = *L. plantarum* (1 × 10^8^ CFU) supplemented group in feed; NC = infected, non-medicated control group.

The total antibody titers in probiotic-supplemented (1 × 10^8^ CFU) group C was higher (*p* ˂ 0.05) than in the infected, non-medicated control group. Among probiotic-supplemented groups, maximum antibody titers were noted in chickens supplemented with 1×10^8^ CFU, followed in decreasing order by the groups supplemented with 1 × 10^7^ CFU and 1×10^6^ CFU. Antibody titers and IgG value of group supplemented with probiotics (1 × 10^8^ CFU in feed) and infected, non-medicated control groups were noted as statistically significant differences (*p* < 0.05), both at day 7 and 14 of post-SRBC treatment (Figure 5a,b).

### 3.5. Serum Enzyme Levels for Toxicity

**Figure 6:**
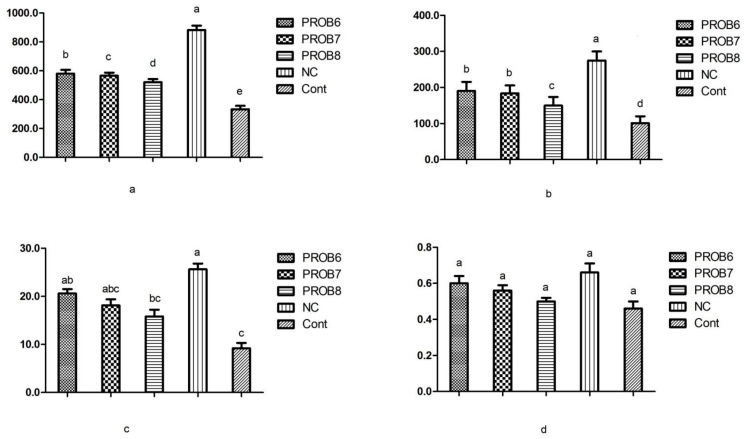
Serum chemistry (LDH, AST, ALT, and creatinine) values (**a**,**b**,**c**, and **d**, respectively) in various treatments of infected chicken groups. Means with different superscripts (a, b, c, d) are significantly different (*p* < 0.05). PROB6 = *L. plantarum* (1 × 10^6^ CFU) supplemented group in feed; PROB7 = *L. plantarum* (1 × 10^7^ CFU) supplemented group in feed; PROB8 = *L. plantarum* (1 × 10^8^ CFU) supplemented group in feed; NC = infected, non-medicated control group; Cont = non-infected, non-medicated control group.

Serum chemistry (ALT, AST, and LDH) values were lower in probiotic-supplemented groups. However, AST and LDH values were significantly different (*p* < 0.05) in all probiotic-supplemented groups in feed, compared with the non-infected, non-medicated, control group (Figure 6).

### 3.6. mRNA Gene Expression

#### 3.6.1. Peptide Transporter 1 (PepT 1) and Antioxidant Enzymes mRNA Expression Levels

**Figure 7:**
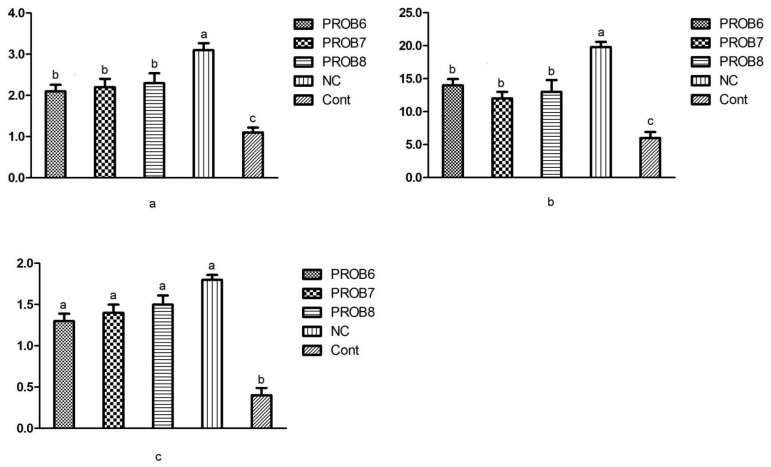
mRNA expression levels of antioxidant enzyme and PepT 1: (**a**) SOD expression level; (**b**) CAT expression level; (**c**) PepT 1 expression level. Means with different superscripts (a, b, c) are significantly different (*p* < 0.05). PROB6 = *L. plantarum* (1 × 10^6^ CFU) supplemented group in feed; PROB7 = *L. plantarum* (1 × 10^7^ CFU) supplemented group in feed; PROB8 = *L. plantarum* (1 × 10^8^ CFU) supplemented group in feed; NC = infected, non-medicated treatment group; Cont = non-infected, non-medicated treated group.

The expression levels of peptide transporter 1 (PepT 1) and alteration in SOD 1 and CAT (antioxidant enzyme) are presented in (Figure 7). Non- infected, non-medicated control group revealed higher mRNA levels of PepT 1 and antioxidant enzymes than the rest of the other groups. In comparison with the infected and untreated birds, probiotic-supplemented birds showed significantly increased (*p* < 0.05) mRNA levels of PepT 1, SOD 1, and CAT (Figure 7a,b,c).

#### 3.6.2. Gene Expression Levels of Tight Junction Proteins

**Figure 8:**
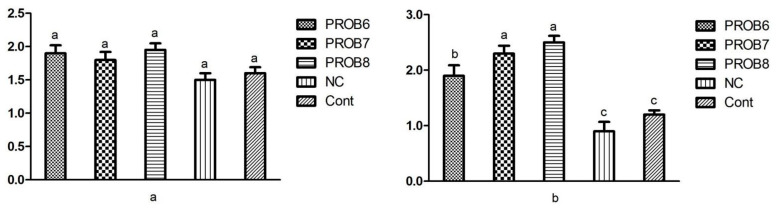
mRNA expression levels of tight junction proteins in different treatment groups: (**a**) CLDN 1 expression level; (**b**) ZO 1 expression level. Means with different superscripts (a, b, c) are significantly different (*p* < 0.05). PROB6 = *L. plantarum* (1 × 10^6^ CFU) supplemented group in feed; PROB7 = *L. plantarum* (1 × 10^7^ CFU) supplemented group in feed; PROB8 = *L. plantarum* (1 × 10^8^ CFU) supplemented group in feed; NC = infected, non-medicated treatment group; Cont = non-infected, non-medicated treated group.

The expression levels of the junctional proteins are represented in Figure 8. A significant upregulation of the mRNA expression levels of ZO 1 was noted in the probiotic-supplemented groups in feed. Moreover, the supplementation with probiotics groups showed a significant (*p* < 0.05) upregulation of the expression of ZO 1 in comparison with the untreated challenged and untreated unchallenged groups (Figure 8b). However, the expression levels of the CLDN 1 showed no significant difference among various treatments groups (*p* > 0.05) (Figure 8a).

## 4. Discussion

Due to drug-resistant issues, the current study was designed to investigate the antioxidant, immunomodulatory, and growth-promoting activities of probiotics. It evaluated the immune-boosting activities of *L. plantarum* based on probiotics in humoral and cellular immune responses. The higher cellular immune response to DNCB was observed in probiotic-supplemented groups. Among probiotic-supplemented groups, the maximum cell-mediated response was noted in the 1 × 10^8^ CFU probiotic-supplemented chicken group, as compared with 1 × 10^7^ CFU and 1 × 10^6^ CFU probiotic-supplemented groups. *L. plantarum* (1 × 10^8^ CFU in feed) showed a significant difference than that of the control group (*p* < 0.05). The capability of probiotics to stimulate natural killer cells, antigen-specific immune cells, and macrophages could be due to the increased cellular immune response [34]. In this study, antibody titers and IgG values of groups supplemented with probiotics (1 × 10^8^ CFU in feed) and infected, non-medicated control group had notable statistically significant differences (*p* < 0.05), both at day 7 and 14 of post-SRBC treatment. Probiotics’ immunomodulatory activities can boost T-cell responses, stimulate phagocytosis, and increase Ig secretion [15]. The prominent immune response in the *Lactobacillus* supplemented groups agrees with a study [35], in which improved antibodies against *E. tenella* infection in the *Pedicoccus*-supplemented bird groups were reported.

Avian coccidiosis leads to intestinal damage by free radicals [36]. Further, reactive oxygen species (ROS) lead to necrosis and modification, including imbalance homeostasis in the intestinal tract of infected birds [37]. Even though birds have immune systems against different pathogens, local antioxidant defense systems are insufficient to prevent the pathogen from damage. Therefore, there is an urgent need to offer adequate supplements within the food having an immunomodulatory and antioxidant potential against the pathogen. In this regard, probiotics have potential as antioxidants due to having functions such as upregulation of antioxidase activities through superoxide dismutase (SOD), downregulation of reactive oxygen species (ROS) producing enzymes, and balancing the beneficial intestinal microbes against oxidative destruction [24]. The antioxidant enzymatic activity among various treatments was also examined in the present study. The study results suggested that the treatment with probiotics in the food resulted in an increased mRNA expression of SOD 1 and CAT, compared with the challenged and untreated control group. Previous studies have also reported that probiotics significantly increase the activities of various antioxidative enzymes against microbial infection [14,24]. Various antioxidant enzymes, including SOD 1 and CAT, are considered important in protecting cells against various stresses by degrading hydrogen peroxide and various superoxide anions [38]. Lower SOD activities in the challenged and untreated control group were probably due to higher production of ROS or low antioxidant performance, resulting in cell destruction and death [36].

Proteins associated with tight junctions such as zonula occludins (ZO), claudin (CLDN), and occluding (OCLN) have essential roles in maintaining the epithelial cell barrier of the intestine. This mechanism is crucial for the defense of host intestines from various pathogenic microbes [39]. In the current study, the group challenged with *E*. *tenella* but untreated significantly decreased the expression of ZO1 and CLDN1 rather than that of other probiotic-supplemented groups. These results are in agreement with earlier studies showing that the treatment with probiotics alone or in combination with herbs significantly improved the barrier function of intestines by enhancing the functional performance of different junctional proteins [40,41]. Therefore, the supplementation of feed with probiotics can prevent the adverse effects of *Eimeria* infection on birds’ growth performance and intestinal functions. Probiotics are also associated with enhancing the antioxidant enzymes’ activity, increasing the regulation of pro-inflammatory cytokines, and tight junctional proteins against pathogens [42].

Different genera of yeast and bacteria-based probiotics, such as *Saccharomyces, Lactobacillus*, *Streptococcus, Pedicoccus,* and *Bifidobacterium*, have shown favorable results against pathogens in various animal models [15,16]. Probiotics are bacterial or yeast-based products that improve the microbial balance of intestines and, directly or indirectly, contribute to positive effects on different animals’ production and reproduction potential and immune systems. Mainly, they are used for the treatment of digestive tract problems and respiratory system issues. In the *E. tenella* challenge, poor FCR and growth performance were possibly due to enormous intestinal damage that occurs with severe infection and causes nutrient malabsorption and homeostasis [6]. Certain nutrients shift from growth to immune response, resulting in a major difference in infected birds’ growth performance from normal birds. Probiotics have essential roles in controlling intestinal diseases [4]. Here, the curative effects of *L. plantarum* on the pathogenesis of *E. tenella* were examined by using different parameters such as fecal scores, OPG, and FCR. The positive effects of bacteria and yeast-based probiotics against *Eimeria* parasites have also been reported in a previous study [15]. Our trial focused on the effects of *L. plantarum* against the *E. tenella* challenge, unlike past research. However, supplementation with probiotics does not significantly improve *Eimeria* infected chicken immunity and body weight [4,43]. Differences may be due to different parameters that may amendment the potential of probiotics, such as probiotics preparation methodologies, the difference in probiotics strains (bacteria, yeast), and probiotics dosage. Other contributors may be environmental stress (temperature), bird age, diet plan, and poultry farm hygienic conditions [43,44].

The oocysts per gram count were considerably lower in chicken groups supplemented with *L. plantarum*. Corroborating our results, *Lactobacillus* supplemented broilers shed fewer oocysts than the *E. acervulina*-infected group [45]. Lower oocysts count may be due to increased production and performance of CD4+ and CD8+ T lymphocytes in the probiotic-supplemented birds [4]. The fewer oocysts and fecal score can also be correlated to probiotics, which may improve intestinal growth by enhancing host immunity, acting as antioxidants, balancing beneficial microbes, and competitive exclusion of pathogens [6,14]. The oocyst reduction, fewer fecal scores in dropping, and improved FCR may be due to improved hemopoietic effects of probiotic-supplemented groups, compared with the infected, non-medicated group, as evinced by a previous study [46].

## 5. Conclusions

The concept of using probiotics as substitutes for antiparasitic drugs is a promising approach to controlling coccidiosis, as shown by a recent trial. Due to drug resistance, the use of probiotics is of great importance. Anticoccidial effects of probiotics were demonstrated in reducing pathogenic impacts by acting as antioxidants and enhancing host immunity, as well as their essential role in improving the integrity of the digestive route. Further experiments are necessary to examine probiotics exact mode of action with different strains and doses of probiotics. Research evaluating omics (mostly genomics, transcriptomic, proteomics, and metabolomics) and gene expression within immune tissues, microbial profiles, histological discrepancy, and other assessable parameters will further confirm the mode of action of probiotics.

## Figures and Tables

**Table 1:** Primers used in this experiment for the assessment of mRNA expression levels.

Genes		Primer Sequence (5′–3′)	Gene Bank
CAT	F	GAGGAACCCTCAGACTCATTTG	NM_001031215.2
	R	CCATCAGGAATACCACGATCAC	
SOD 1	F	AGATGGCAGTGGGAAATGAG	NM_205064.1
	R	ACTCAAGACAGCAGAGTAGTAATG	
PepT 1	F	CCCCTGAGGAGGATCACTGTT	KF366603.1
	R	CAAAAGAGCAGCAGCAACGA	
CLDN 1	F	ACTCCTGGGTCTGGTTGGT	AY750897.1
	R	CAGGTCAAACAGAGGTACAGG	
ZO 1	F	CTTCAGGTGTTTCTCTTCCTCCTC	XM_413773
	R	CTGTGGTTTCATGGCTGGATC	
β-actin	F	GAGAAATTGTGCGTGACATCA	L08165
	R	CCTGAACCTCTCATTGCCA	

**Table 2:** Fecal score in chickens (*n* = 6) with experimentally induced coccidiosis in different treated groups.

Groups	Day 5th	Day 6th	Day 8th
**PROB6**	2.50 ± 0.33 ^b^	2.83 ± 0.27 ^b^	2.33 ± 0.54 ^b^
**PROB** **7**	2.33 ± 0.54 ^b^	2.66 ± 0.14 ^b^	2.00 ± 0.51 ^b^
**PROB** **8**	1.67 ± 0.14 ^c^	1.67 ± 0.15 ^c^	1.33 ± 0.21 ^c^
**NC**	3.33 ± 0.15 ^a^	3.50 ± 0.16 ^a^	3.33 ± 0.15 ^a^
**Cont**	^_^	^_^	^_^

Means with different superscripts (a, b, c) inside a column (Table 2) are significantly different (*p* < 0.05). PROB6 = *L. plantarum* (1 × 10^6^ CFU) supplemented in feed; PROB7 = *L. plantarum* (1 × 10^7^ CFU) supplemented in feed; PROB8 = *L. plantarum* (1 × 10^8^ CFU) supplemented in feed; NC = infected, non-medicated treatment group; Cont = non-infected, non-medicated treated group.

## Data Availability

The data presented in this study are available within the article.
